# Supplementary material for: Feeding efficiency of two coexisting nectarivorous bat species (Phyllostomidae: Glossophaginae) at flowers of two key-resource plants
Source: PLoS One. 2024 Jun 26;19(6):e0303227. doi: 10.1371/journal.pone.0303227 (PMC11207168; doi:10.1371/journal.pone.0303227)
Supplement: Attachment — Submitted filename: Response to reviewers.pdf [file pone.0303227.s005.pdf]

Dear Dr. Becker,

we received the reviews for our manuscript “Feeding performance of nectar-feeding bats (Phyllostomidae: Glossophaginae) indicates that familiarity with flowers shapes resource utilization” and we thank you and the reviewers for the helpful and constructive comments on our work. We have edited our manuscript to address the concerns.

We hope that we were able to address the issues satisfyingly and that the manuscript is now suited for publication in PLOS ONE,

Sincerely,

On behalf of all authors,

Jan Philipp Bechler

Academic Editor

Greater clarity in articulating hypotheses and predictions in the introduction. Lines 117-128 come close, and so some modification in wording should help here, particularly L124-128.

We modified the hypothesis and the predictions and hope that this resulted in better clarity (lines 131-149).

Specific statements about sample size must be included.

We admit that in some parts the sample size was not clearly stated. We added sample size where missing.

Reviewer 2 also commented on the need to include a LMM instead of LM; from my reading of L234-249, it does appear LMMs were used here, given the mention of fixed and random effects, so the authors should be sure to describe these analyses as "linear mixed effects models" rather than "linear regression models" to be as specific as possible and avoid confusion.

Indeed, we used the more general term "linear regression" but calculated in fact a LMM. In response to reviewer 2 we eliminated this expression from the method description to avoid confusion about the used model type and stated that we used "linear mixed effect models from the lme4-package" (line 266).

Lastly, I would also suggest the authors include confidence intervals (Figure 1) and raw data (rather than only means and confidence intervals in Figure 5) in their visualizations.

We assume that confidence intervals were suggested for Figure 3 (and not for Figure 1). Confidence intervals were added. For Figure 5 we changed the plot type from mean +/- SE to boxplots to better represent the distribution of raw data.

1. Please ensure that your manuscript meets PLOS ONE's style requirements, including those for file naming. The PLOS ONE style templates can be found at https://journals.plos.org/plosone/s/file?id=wjVg/PLOSOne_formatting_sample_main_body.pdf and

The manuscript was revised and edited according to the PLOS ONE's style requirements.

https://journals.plos.org/plosone/s/file?id=ba62/PLOSOne_formatting_sample_title_authors_affiliations.pdf

The title, authors and affiliations now meet the style requirements. We changed the e-mail address of the corresponding author.

2. Please include a complete copy of PLOS' questionnaire on inclusivity in global research in your revised manuscript. Our policy for research in this area aims to

improve transparency in the reporting of research performed outside of researchers' own country or community. The policy applies to researchers who have travelled to a different country to conduct research, research with Indigenous populations or their lands, and research on cultural artefacts. The questionnaire can also be requested at the journal's discretion for any other submissions, even if these conditions are not met. Please find more information on the policy and a link to download a blank copy of the questionnaire here: https://journals.plos.org/plosone/s/best-practices-in-research-reporting. Please upload a completed version of your questionnaire as Supporting Information when you resubmit your manuscript."

A copy of PLOS' questionnaire on inclusivity in global research is included and attached as Supporting Information.

3. Please include your full ethics statement in the 'Methods' section of your manuscript file. In your statement, please include the full name of the IRB or ethics committee who approved or waived your study, as well as whether or not you obtained informed written or verbal consent. If consent was waived for your study, please include this information in your statement as well.

We included a full ethics statement into the 'Methods' section containing the information of the local approving authority and the respective permit numbers.

4. Please include captions for your Supporting Information files at the end of your manuscript, and update any in-text citations to match accordingly. Please see our Supporting Information guidelines for more information: http://journals.plos.org/plosone/s/supporting-information.

Captions for the Supporting Information files were attached at the end of our manuscript and we updated the in-text citations.

5. Please review your reference list to ensure that it is complete and correct. If you have cited papers that have been retracted, please include the rationale for doing so in the manuscript text, or remove these references and replace them with relevant current references. Any changes to the reference list should be mentioned in the rebuttal letter that accompanies your revised manuscript. If you need to cite a retracted article, indicate the article's retracted status in the References list and also include a citation and full reference for the retraction notice.

The reference list was reviewed to ensure that it is complete and correct.

Reviewer #1 (Brock Fenton)

First, the authors should clearly articulate the hypothesis that guided the work. Then they should identify specific predictions they can test with their data. While the authors cruise close to this approach, they could be more specific and clearly distinguish between hypothesis and prediction.

Thank you for your observation. We addressed the issue and reviewed the hypotheses and predictions to increase clarity.

I admire the authors' approach and candor concerning which of their expectations were supported (or not supported) by the data). Often things did not emerge as expected. What does this mean? It could mean that the predictions are not sharp enough, perhaps suggesting that some underlying aspect of the system they use is not correctly presented. Alternatively the bats may be generalist enough to behave in unexpected ways. Evidence from gut biome and isotope research reveals that classifications of bats by diet are misleading at best, and perhaps confusing as well. There also is evidence that at least Glossophaga soricina relies much more on insects as food than suggested by their designation as nectar-feeders.
I think that the data from gut biomes and isotopes are relevant to understanding the current situation.

We intentionally stuck with our initial hypotheses with which we started the project and did not just adjust our hypothesis to our findings, although we admittedly learned a lot about our study species in the progress of the project (which is ultimately the purpose of science ;-)). It is true that the *Glossophaga* species are considered as generalist, however we are convinced that the generalistic feeding mode is not an inherit trait of this species, but rather a strategy to cope with local or temporal nectar scarcity. These bats show distinct morphological and physiological adaptations to nectarivory and always prefer nectar when it is sufficiently available and only supplement their diet with other sources (insects, fruits) when nectar is scarce. For the area of La Selva, this is nicely demonstrated by seeds of *Piper auritum* in the feces of *G. commissarisi* (Tschapka, 2005). These infructescences are available all year-round but seeds are only evident in feces of *G. commissarisi* during the time of low flower abundance. Thus we are confident that the classification of *G. commissarisi* as nectarivorous bat species is valid for our study area. It is crucially important to collect year-round data for a specific site to fully understand the meaning of certain food resources for the respective species. Data from only short periods (like in most isotope or microbiome analyses) only provide temporal snapshots and thus are not suitable for making general statements on the degree of specialization of a species. For La Selva, we thankfully have these long-term data and have solid background information about the bats and their resources over the annual cycle (e.g., Tschapka 2004, 2005, Becker et al. 2010, Rothenwöhrer et al 2011). We now also emphasize this argument in our manuscript (lines 109 – 122).

In short, this is a neat paper. The authors have done an excellent job, especially in getting others (at least me) to think about the situation in different ways.

Thank you!

Reviewer #2 (anonymous)

Major points:

1 – The first regards to the more precise use of the literature and the lack of hypothesis that do not help understanding the data analysis and results. It is not clear why authors are comparing and focusing on the explanation of many other points if their hypothesis should be to explore the drinking efficiency (volume of extracted nectar) in relation to flower orientation. If you could delimitate and focus on a single research question and

their associated predictions it would be easy to understand and interpret the results. I suggest authors to follow the guidelines presented here to help in their revision: https://conservationbytes.com/2012/10/22/how-to-write-a-scientific-paper/ . https://www.springernature.com/br/authors/campaigns/writing-a-manuscript/titles-abstracts-keywords https://journals.plos.org/ploscompbiol/article?id=10.1371/journal.pcbi.1005619 https://www.nature.com/articles/d41586-018-02404-4

Thank you for your observation! You helped us to understand where our manuscript might be misleading. Actually, we did not intend to study the role of flower orientation on the feeding performance of nectar-feeding bats. Although we discuss this as one of the main explanations for our findings, it is not the main aim of the study. We happily encourage further research on this topic as we think this would be a great starting point for further studies. So far, only little is known about the role of floral orientation. While it might be a substantial factor driving the interaction, the detailed investigation of floral orientation would have exceeded the scope of this study and thus was included only as a part of the discussion. However, thanks to your comment, we understand that the argument of flower orientation was too present throughout the manuscript, thus giving the impression that this was the main objective of this study. In the revised manuscript we therefore restricted the emphasis on the differential flower orientation between the two focal plant species to the Discussion, to help emphasizing the main purpose of our study.

2 – The second major point regard to methods, it is not clear for me the total number of each bat species (n=) you used in each experiment, you need to describe the experiments and data collection in more, much more, detail. I added detailed comments below.

Thank you for pointing this out. We admit that the sample sizes were not always clearly stated and we added sample sizes to help clarification where needed.

3 – The third regards to the statistical analysis, it is not clear if the authors performed a linear regression or a fixed effects model. I actually recommend a GLMM (generalized linear mixed model, or a Linear Mixed Model, LMM) to estimate the effect of bat´s feeding specialization (specialist, generalist) on drinking efficiency (extracted nectar volume) and the different variables of interest (bat size, visit duration, flower species, flower source -natural, artificial-, flower size, flower orientation and flower arrangement) of the single bat individuals (n=?). There are many good sites in the net explaining the use of GLMMs, for example: https://www.researchgate.net/publication/221995574_Generalized_Linear_Mixed_Models_A_Practical_Guide_for_Ecology_and_Evolution https://www.frontiersin.org/subjects/generalized-linear-mixed-model

Indeed we used the more general term “linear regression model”, while actually we did specifically an LMM. Based on you comment, we eliminated this expression from the method description to avoid confusion about the used model type and stated clearly that we used “linear mixed effect models from the lme4-package” (lines 266).

4 – Finally, I think what you have collected is really interesting, but you need to eliminate the lack of clarity for the reader to follow your results and discussion sections by delimitating your research question. Detailed comments below.

Thank you, we really appreciate your helpful comments. We hope that with our revisions we increased the clarity of our manuscript.

Detail feedback

I suggest to change the title to make it clearer:
Bats nectar extraction efficiency of the specialist Hylonycteris underwoodi and the generalist Glossophaga commissarisi (Phyllostomidae:Glossophaginae) in relation to flower orientation.

As explained above, we restricted the emphasis on floral orientation to the discussion as its only one aspect explaining our findings. However, we agree that the title needed some improvement and changed it to "Feeding performance of nectar-feeding bats (Phyllostomidae: Glossophaginae) at flowers of two key-resource plant species".

Abstract
L-(29-34): extensive background and did not include de gap.

Based on your comment, we now specifically added a remark on the lack of studies on real flowers for this kind of studies (lines 32 - 33).

L-42: the most relevant results were not included.

We now added the most relevant results (lines 45 – 47), which should now help to better understand the following conclusion.

L-(42-45): it is not clear what you relied on to stand this conclusion.

In combination with the main results we hopefully now clarified the interpretation of the outcome of the study.

Introduction
In general, it is better to give information from general to specific topics, from bat´s food selection in field in general, and then to the generalists and specialists nectar feeding bats. Regarding the use of natural nectar feeding sources, and differences within specialist vs generalist bat species there is important literature missing to be included.

We added further information on the bat species and their food selection in natural habits to the Introduction to help better understand the framework of our study (lines 93 – 122).

L-72: important baselines. However,

Thank you. We changed our writing accordingly. Splitting this sentence into two definitely increases readability.

L-(82-88): You don`t talk about previous studies that address factors that bats select or not in food, neither in the field, nor in experimental conditions. Nor do you mention previous studies comparing whether specialist bats select the same as generalists or not, and why. Explain it here and then directly connect them with your aims.

We hope that our changes now emphasize the actual lack of such studies and provide sufficient (long-term) background information on the general resource situation of nectar-feeding bats in the La Selva area that is necessary to understand the frame of our study and why this is a great and unique place to do exactly this kind of study there. To our very best

knowledge, there are only very few studies focusing on nectar-feeding bats of different degrees of specialization.

Methods

I suggest to include studied species information in this section and to give more specific details about plant species and bat species as the total number of individuals used in experiments, and other relevant information about bats' ecology and experiments. Also, I suggest to only include data regarding to natural flowers experiments to simplify the main message of the paper.

We added further information on the species into the Introduction and we are confident that the provided information now suffices to understand the ecology of the focal species.

We adjusted the description of the Methods, especially we emphasized that only one bat at a time was used in the experiments as we understand that it was not clearly stated before (lines 168 – 176).

You suggested to only including data on real flowers in the paper. Thus far, the majority of studies on the feeding performance of nectar-feeders were conducted with artificial flowers (ideal scenarios) and the obtained insights were then transferred to natural systems. Our study provides now also the opportunity to test the transferability of such approaches as we can directly compare it with the feeding performance at real flowers. As we unexpectedly observed a substantially higher caloric yield at real flowers compared to the artificial setup in *H. underwoodi* we are convinced that this result is valuable information, and emphasizes the need for more studies with real flowers in order to better understand the complex spectrum of animal-plant interactions.

L-(136-145): These lines do not correspond to Field Site description. Add another subtitle regarding to Bats Collection.

We appreciate your observation and changed the subtitle to "Field site and bat capture" (line 153).

L-(same lines): Did bats were collected the same day of the experiments or did they were maintained in captivity previous days before the experiments? How many? In which conditions? How did you collected bats?

We clarified the description of the procedure of the experiments following bat capture, hoping to eliminate any potential uncertainties. We acknowledge that the original version did not state this clear enough (lines 159 – 165) and emphasize now that we only used one individual at a time during our experiments.

L-(149-151): Does 16% of nectar total concentration corresponds to the natural nectar mean concentration of both plant species? Why did you choose to use this nectar concentration and sugar composition?

We provide now further information on natural nectar concentration of chiropterophilous plant species in general (lines 168 – 171) and for the focal plant species specifically (lines 178 - 181) as well as a justification for the use of honey water for the experiments (lines 171 – 176). 16 % corresponds to the mean sugar concentration of nectar from chiropterophilous flowers (von Helversen 1993). Further, 16 % falls within the natural nectar sugar

concentration range for both of our focus plant species, and thus we chose 16 % as the standard nectar concentration for our experiments.

L-(same lines): Did you use the same flower species in the same arrangement? Why don't you changed in another experiment the arrangement among plant species to really test the effect of flower species and/or spatial flower arrangement? How did you control for position effect?

Thanks to your observation, as we mentioned above, we eliminated the strong emphasis on the flower orientation outside the discussion, as the main objective was not to address the flower orientation, but real flowers in their natural appearance.

L-188: Why did you choose to use glucose and fructose and not sucrose? You don't talk about natural nectar sugar composition and total concentration of plant species.

Honey mainly consists out of the two hexoses glucose and fructose (for about equal parts). For what it is known from several plant families, this also applies to chiropterophilous flowers. We addressed this issue now in lines 171 – 173. Further, from our captive bat colony we know that a diet from 16 % honey-water can maintain a healthy bat-population. Also honey-water – in contrast to a pure sucrose solution - has a sweet odor and from our experience we know that this helps bats to locate the offered food resources in novel, experimental situations.

L-197: Why is it necessary to compare an artificial design with a natural stage? What do you expect to obtain? Why don´t you only include results obtained with the most natural field conditions?

Many studies on the feeding performance of nectar-consumers were conducted with "artificial flowers", i.e., often just glass tubes with an *ad-libitum* availability of nectar. These settings constitute a kind of ideal scenario, as the nectar uptake is not limited by further factors. These studies provide valuable baselines about basic principles of nectar drinking. However, under natural conditions animals only rarely (or even never) face such ideal conditions, as flowers only usually contain only limited nectar volumes and further internal floral structures might impede access to floral nectar (and thus may extend the duration of the visit and increase pollination probability for the plant). Thus, we see a strong need for comparative studies to explore to which extent the insight gained from artificial settings correspond to the natural world. According to you comment, we further emphasized the need for such comparative studies (lines 226 – 233).

L-(216-220) If you are testing bat´s feeding efficiency why it is important to include pollen load in this paper?

In our study, we discuss the observed differences in feeding performance between the two bats species in the context of the ecological conditions these bats face in the study area of La Selva Biological Station in Costa Rica. The species-specific resource availability and - use over the course of a year are rather valuable background information. The long-term pollen data illustrates the pronounced use of both plant species during the annual cycle and confirm the role of both plant species as key resources for the resident nectar-feeding bats in the study area.

Figures
No figure legends are provided.

We agree that inclusion of figure legends improves the understandability. However, in manuscript preparation we followed the guidelines for PLOS ONE and accordingly the figure legends were provided within the text.
