# Supplementary material for: Feeding efficiency of two coexisting nectarivorous bat species (Phyllostomidae: Glossophaginae) at flowers of two key-resource plants
Source: PLoS One. 2024 Jun 26;19(6):e0303227. doi: 10.1371/journal.pone.0303227 (PMC11207168; doi:10.1371/journal.pone.0303227)
Supplement: Attachment — Submitted filename: Response to Reviewers.pdf [file pone.0303227.s007.pdf]

Dear Dr. Becker,

we received the reviews for our manuscript "Feeding performance of nectar-feeding bats (Phyllostomidae: Glossophaginae) indicates that familiarity with flowers shapes resource utilization" and we thank you and the reviewers for the helpful and constructive comments on our work. We have edited our manuscript to address the concerns.

We hope that we were able to address the issues satisfyingly and that the manuscript is now suited for publication in PLOS ONE,

Sincerely, On behalf of all authors,

Jan Philipp Bechler

Reviewer #2:

In most of the answers to my previous requests in the last revision, it seems that authors responded and completely attended to them. However, they do not include the modified words, or punctually the requested changes in the answer to reviewers, and sometimes, they did not include the lines, and when I read the whole section I find that changes are not included. Please attend in detail to my concerns and be more specific since many of my previous requests were not met in detail.

As in the previous revision, the aims are not connected with what you are fully explaining in the introduction. You talk a lot about plants and your study is about bat selection, you are not talking about this, and if you are measuring feeding efficiency why don't you talk about it deeply and explain which traits on nectar and feeding resources bats rely their food selection?
Clarify what is feeding performance.
Thank you for you comment and feedback on our manuscript. To clarify, our study did not specifically target food selection, but rather investigated the role of plants in bats' foraging behavior and especially their feeding efficiency, but also hovering duration and nectar uptake (summarized as "feeding performance"; see comment below). Besides plants traits we also discuss the role of bats' familiarity with and dependence on these plants in achieving this feeding performance. Regarding this topics, there are currently few studies addressing this aspects with real instead of artificial flowers. However, with this the present study we hope to contribute to close this gap in knowledge and also encourage further work on this fascinating topic.

How did you measure feeding performance? Since you say you calculated nectar extraction efficiency and caloric uptake are them all the same? If not, why are these variables not included in your objectives and hypotheses? Please clarify first concepts in the introduction and here in methods the way you quantified each variable, cause it seems that methods are not connected with the introduction.
We apologize for the confusion in our previous response. Let us address your questions and provide more clarity on the issue. We agree that the use of the term "feeding performance" might be confusing to the reader. This expression is intended to include the different variable we addressed in this study, i.e. hovering duration, nectar uptake, feeding efficiency; thus being a more general expression. We specified this in **L35 & L36** in the abstract, in **L98 & L99** in the introduction and **L216 & L217** in the methods.

Again, for me is not clearly justified here why performing experiments in both, natural and experimental conditions. what is the difference in the information provide by each approach?

We further clarified the justification for this aspect of the study (**L171 – L173**). Collecting data on feeding behavior at real flowers now gives us the unique opportunity to compare the artificial approach (what was usually done in the past) to the natural condition. This provides insight into the validity of the information gained from experimental with artificial flowers.

Results are confusing and not easy to follow, and I can`t find the most important contribution of the study. Please follow the same order in studied variables along the whole manuscript.

We made sure that we followed the same order of variables for both sections (comparison between bats and comparison between plant species) and also maintain the same order in the discussion. However, as we compare both, differences between bat species and differences between plant species, this dual approach requires occasionally deviating from this order, with the intention to keep up the logical flow in the discussion.

Discussion section is quite long and imprecise, the most important results are not well discussed.

Again, thank you for the time you took to review our paper and we appreciate your feedback. We understand you concerns regarding length and precision of the discussion section. However, given the complexity of the results and after careful consideration, we think that the current version provides a thorough and comprehensive discussion of our results, including their significance and implications. We would appreciate if you could provide us with a bit more specific feedback regarding which important results are not well discussed, as this would enable us to address your concerns more specifically.

ABSTRACT

In the abstract section any of the 3 major changes I asked for in the previous revision were attended. They were: extensive background and did not include the gap, the most relevant results were not included and it is not clear what you relied on to stand this conclusion.

L. 29-37 Very large and general background, please be more accurate to your study question.

We shortened and streamlined this part substantially.

L.38 It is not yet clear stated how did you measure feeding performance.

L35 & L36: Feeding performance, as a general term for the measured aspects (nectar uptake, hovering duration and the resulting feeding efficiency) is now stated in the abstract.

L 40-45 Please rewrite these sentences since you included mixed sections, hypothesis and methods, making the abstract very difficult to catch the most important message. It is not

clear what and how you measure, what and how you compare, between plant species? Between two bat species? Between selection of the specialist and generalist bats?
L38 & L39: Thank you for your helpful comment. We further specified what we measured (see comment above) as well as what we compared. We also aimed to make the difference between methods and hypotheses more clear.

L.42 Bromeliad?
Werauhia gladioliflora is a member of the Bromeliaceae family and here we aimed to provide brief systematic information on the studies plant species to the interested reader.

L.45 The results section did not match to what I would expect reading the previous lines, as I would expect to see results for 2 bat species interacting with two plant species, and then the comparison between specialist vs generalist bats feeding performance. Instead, you only included to present results for *H. underwoodi* at only one plant species (*M. neuranthum*). If no differences with the generalist bat species and the other plants were found, please state it clearly here.
L43-L49: We revised the commented part of the abstract and now clearly mention the results on both bats species and on both data from real flowers as well as results from the comparison between real and artificial flowers. We hope we now provide a satisfactory level of information.

L. 47 How did you arrive to say this?
Please see comment above.

L.50 You are not providing a general closing with your most important finding.
L68-L72: A general closing is now provided in at the end of the section.

INTRODUCTION
L. 138 In which previous studies are you relying your hypotheses? Further, in the previous paragraphs you do not talk about on which you included in your hypotheses.M
We agree that it is important to provide a clear understanding of the existing literature and how our work fits into it. However, our study investigates a novel topic that has not been previously explored in the literature. Therefore, we were not able to refer to any previous studies in developing our hypothesis, but simply base this assumption on the nectar-rich nature of the *Werauhia* flowers as it is reasonable to assume that larger nectar-volumes in a flower facilitate a higher feeding efficiency. We now specify this notion in this part, hoping to have clarified our hypothesis. We have taken your comment into consideration and clarify how our hypotheses were developed.

MATERIAL AND METHODS

L.227 experiments
Thank you for your correction. We changed experiment to the plural form.
