# Supplementary material for: Feeding efficiency of two coexisting nectarivorous bat species (Phyllostomidae: Glossophaginae) at flowers of two key-resource plants
Source: PLoS One. 2024 Jun 26;19(6):e0303227. doi: 10.1371/journal.pone.0303227 (PMC11207168; doi:10.1371/journal.pone.0303227)
Supplement: Attachment — Submitted filename: Revision PONE-D-21-39791 PLOSONE.docx [file pone.0303227.s006.docx]

In most of the answers to my previous requests in the last revision, it seems that authors responded and completely attended to them. However, they do not include the modified words, or punctually the requested changes in the answer to reviewers, and sometimes, they did not include the lines, and when I read the whole section I find that changes are not included. Please attend in detail to my concerns and be more specific since many of my previous requests were not met in detail.

As in the previous revision, the aims are not connected with what you are fully explaining in the introduction. You talk a lot about plants and your study is about bat selection, you are not talking about this, and if you are measuring feeding efficiency why don’t you talk about it deeply and explain which traits on nectar and feeding resources bats rely their food selection?

Clarify what is feeding performance.

How did you measure feeding performance? Since you say you calculated nectar extraction efficiency and caloric uptake are them all the same? If not, why are these variables not included in your objectives and hypotheses? Please clarify first concepts in the introduction and here in methods the way you quantified each variable, cause it seems that methods are not connected with the introduction.

Again, for me is not clearly justified here why performing experiments in both, natural and experimental conditions. what is the difference in the information provide by each approach?

Results are confusing and not easy to follow, and I can`t find the most important contribution of the study. Please follow the same order in studied variables along the whole manuscript.

Discussion section is quite long and imprecise, the most important results are not well discussed.

ABSTRACT

In the abstract section any of the 3 major changes I asked for in the previous revision were attended. They were: extensive background and did not include the gap, the most relevant results were not included and it is not clear what you relied on to stand this conclusion.

L. 29-37 Very large and general background, please be more accurate to your study question.

L.38 It is not yet clear stated how did you measure feeding performance.

L 40-45 Please rewrite these sentences since you included mixed sections, hypothesis and methods, making the abstract very difficult to catch the most important message. It is not clear what and how you measure, what and how you compare, between plant species? Between two bat species? Between selection of the specialist and generalist bats?

L.42 Bromeliad?

L.45 The results section did not match to what I would expect reading the previous lines, as I would expect to see results for 2 bat species interacting with two plant species, and then the comparison between specialist vs generalist bats feeding performance. Instead, you only included to present results for *H. underwoodi* at only one plant species (*M. neuranthum*). If no differences with the generalist bat species and the other plants were found, please state it clearly here.

L. 47 How did you arrive to say this?

L.50 You are not providing a general closing with your most important finding.

INTRODUCTION

L. 138 In which previous studies are you relying your hypotheses? Further, in the previous paragraphs you do not talk about on which you included in your hypotheses.

MATERIAL AND METHODS

L.227 experiments
