# Supplementary material for: Feeding efficiency of two coexisting nectarivorous bat species (Phyllostomidae: Glossophaginae) at flowers of two key-resource plants
Source: PLoS One. 2024 Jun 26;19(6):e0303227. doi: 10.1371/journal.pone.0303227 (PMC11207168; doi:10.1371/journal.pone.0303227)
Supplement: Attachment — Submitted filename: review PONE-D_21-39791_R2.docx [file pone.0303227.s008.docx]

#### PONE_D_21_39791

#### Feeding performance of nectar-feeding bats (Phyllostomidae: Glossophaginae) at flowers of two key-resource plant species

This is an interesting study that contributes to the knowledge of the use of feeding resources by nectarivore bats. However, I suggest major changes related to its structure, ideas order, and wording.

Introduction

You talk about optimal foraging theory, implications of foraging behaviour, pollinator-plant interactions, plants rewards, nectar uptake, however, it is not clearly defined and delimited what is feeding performance. This is the main concept you should develop in the introduction, which variables are included in this concept, which of them are already studied in natural and artificial conditions and which are not, considering plant species and pollinator types.

What do you expect to see in natural conditions and what in artificial conditions, what are both approaches important to include?

The objective stated the comparison of drinking efficiency, is it ok? What about the other variables in “feeding performance”?; if so, then this must be specified in the tittle, it is confusing, or either stay all feeding performance variables you are going to test here. Are the same feeding performance variables going to be tested in both, natural and artificial conditions?

You stated hypotheses, however, you did not included citations on which you rely them.

I suggest that the ideas be organized as follows: First paragraph- Define feeding performance and which variables are considered in this concept; Second paragraph- State the background, in-depth review of the literature, addressing the state of the art of each of the feeding performance variables, under natural and artificial conditions by pollinator type and plants. End the paragraph talking about bats background and gaps. Third paragraph- 1) Clearly stay the aim of the study. 2) Clearly justify why you are using natural and artificial assays. Explain here why you measure hovering duration (energetic investment), extracted volume (caloric uprake) and standardized nectar extraction efficiency. 3) Clearly justify why you are using two bat species and two plant species.

Material and methods

193-194 It says “We aimed to quantify the feeding performance (i.e. hovering duration, nectar uptake, and feeding efficiency)” which do not match with what you stated in your objective.

Explain in the same order how did you measure each of: 1) hovering duration, 2) extracted volume and 3) standardized nectar extraction efficiency.

L 258-262 It is not clear why including pollen samples in bats, since you did not say anything about in the introduction, objective and hypotheses. How important is it in feeding performance? If so, you should include it throughout the introduction.

Results

Present results as:

Natural flowers

1) Energetic investment, 2) Caloric uptake and 3) standardized nectar extraction efficiency.

Artificial flowers

2) Energetic investment, 2) Caloric uptake and 3) standardized nectar extraction efficiency.

In the case tested variables are the same in both, natural and artificial flowers. Is confussing since in artificial flowers it says “standardized nectar uptake” “standardized caloric yield” “caloric net yield”. It is not clear at all if tested variables are the same in natural and artificial flowers, is o, please use the same terms throughout the manuscript. If not please clearly explain in the last paragraph of the introduction as suggested and in the methods section.

Discussion

Discussion is a confusing and quite long section. Please present the discussion section in the same order than in results. Stay the most important findings one by one. For each one, explain your finding, physiological and ecological implications, then compare your finding with previous literature.

Conclusion

Only refer to highlight your most important findings.
