# Supplementary material for: Feeding efficiency of two coexisting nectarivorous bat species (Phyllostomidae: Glossophaginae) at flowers of two key-resource plants
Source: PLoS One. 2024 Jun 26;19(6):e0303227. doi: 10.1371/journal.pone.0303227 (PMC11207168; doi:10.1371/journal.pone.0303227)
Supplement: Attachment — Submitted filename: Response to Reviewers.docx [file pone.0303227.s009.docx]

#### PONE_D_21_39791 Reviewer's Responses to Questions

**Comments to the Author**

**1. If the authors have adequately addressed your comments raised in a previous round of review and you feel that this manuscript is now acceptable for publication, you may indicate that here to bypass the “Comments to the Author” section, enter your conflict of interest statement in the “Confidential to Editor” section, and submit your "Accept" recommendation.**

Reviewer #1: (No Response)

Reviewer #2: (No Response)

**2. Is the manuscript technically sound, and do the data support the conclusions?**

The manuscript must describe a technically sound piece of scientific research with data that supports the conclusions. Experiments must have been conducted rigorously, with appropriate controls, replication, and sample sizes. The conclusions must be drawn appropriately based on the data presented.

Reviewer #1: Yes

Reviewer #2: Partly

**3. Has the statistical analysis been performed appropriately and rigorously?**

Reviewer #1: N/A

Reviewer #2: Yes

**4. Have the authors made all data underlying the findings in their manuscript fully available?**

The [PLOS Data policy](http://www.plosone.org/static/policies.action#sharing) requires authors to make all data underlying the findings described in their manuscript fully available without restriction, with rare exception (please refer to the Data Availability Statement in the manuscript PDF file). The data should be provided as part of the manuscript or its supporting information, or deposited to a public repository. For example, in addition to summary statistics, the data points behind means, medians and variance measures should be available. If there are restrictions on publicly sharing data—e.g. participant privacy or use of data from a third party—those must be specified.

Reviewer #1: Yes

Reviewer #2: No

**Response JPB:** All the necessary data have indeed been uploaded as part of the supplementary materials accompanying our submission (see S1_Data). Reviewer #1 has also acknowledged that all data has been provided. If there are still any specific concerns please do not hesitate to let us know. We are committed to providing all the necessary information to support a thorough evaluation of our work.

**5. Is the manuscript presented in an intelligible fashion and written in standard English?

PLOS ONE does not copyedit accepted manuscripts, so the language in submitted articles must be clear, correct, and unambiguous. Any typographical or grammatical errors should be corrected at revision, so please note any specific errors here.**

Reviewer #1: Yes

Reviewer #2: No

**Response JPB:** In regard to the feedback concerning the quality of the English language in our publication, we understand the importance of adhering to language standards in scientific communication. We have once more tried to shorten and improve the languange and hope that it now meets the approval of both referees.

**6. Review Comments to the Author**

Please use the space provided to explain your answers to the questions above. You may also include additional comments for the author, including concerns about dual publication, research ethics, or publication ethics. (Please upload your review as an attachment if it exceeds 20,000 characters)

**Reviewer #1:**

This manuscript is overwhelming because it presents a great deal of information without a **clear context**.
**What is the hypothesis that underlies the work?** What **specific (numbered) predictions** arising from the hypothesis that the authors can test with the data. Adding this information will dramatically increase the impact of the work by making it clearer to the readers, whether expert of interested colleague.

**Tighten the writing - make clear statements**. One subject per sentence, subject at the beginning of the sentence. Make sure of **the spelling and word choice.**

I think that this manuscript has the potential to be a classic. But both previous reviewers have suggested changes that have not clearly been used to advantage by the authors. Make sure that you need to present all of the details ... **consider omitting tangential material to improve the focus** of the manuscript.

Brock Fenton

**Reviewer #2:**

This is an interesting study that contributes to the knowledge of the use of feeding resources by nectarivore bats. However, I suggest major changes related to its structure, ideas order, and wording.

Introduction
You talk about optimal foraging theory, implications of foraging behaviour, pollinator-plant interactions, plants rewards, nectar uptake, however, it is not clearly defined and delimited what is feeding performance. This is the main concept you should develop in the introduction, which variables are included in this concept, which of them are already studied in natural and artificial conditions and which are not, considering plant species and pollinator types.

What do you expect to see in natural conditions and what in artificial conditions, what are both approaches important to include?

The objective stated the comparison of drinking efficiency, is it ok? What about the other variables in “feeding performance”?; if so, then this must be specified in the tittle, it is confusing, or either stay all feeding performance variables you are going to test here. Are the same feeding performance variables going to be tested in both, natural and artificial conditions?

You stated hypotheses, however, you did not included citations on which you rely them.

I suggest that the ideas be organized as follows:

1. First paragraph- Define feeding performance and which variables are considered in this concept;
2. Second paragraph- State the background, in-depth review of the literature, addressing the state of the art of each of the feeding performance variables, under natural and artificial conditions by pollinator type and plants. End the paragraph talking about bats background and gaps.
3. Third paragraph-
   a. 1) Clearly stay the aim of the study.
   b. 2) Clearly justify why you are using natural and artificial assays. Explain here why you measure hovering duration (energetic investment), extracted volume (caloric uprake) and standardized nectar extraction efficiency.
   c. 3) Clearly justify why you are using two bat species and two plant species.

Material and methods
193-194 It says “We aimed to quantify the feeding performance (i.e. hovering duration, nectar uptake, and feeding efficiency)” which do not match with what you stated in your objective.
Explain in the same order how did you measure each of: 1) hovering duration, 2) extracted volume and 3) standardized nectar extraction efficiency.

L 258-262 It is not clear why including pollen samples in bats, since you did not say anything about in the introduction, objective and hypotheses. How important is it in feeding performance? If so, you should include it throughout the introduction.

Results
Present results as:
Natural flowers

1) Energetic investment,

2) Caloric uptake and

3) standardized nectar extraction efficiency.

Artificial flowers
2) Energetic investment,

2) Caloric uptake and

3) standardized nectar extraction efficiency.

In the case tested variables are the same in both, natural and artificial flowers. Is confussing since in artificial flowers it says “standardized nectar uptake” “standardized caloric yield” “caloric net yield”. It is not clear at all if tested variables are the same in natural and artificial flowers, is o, please use the same terms throughout the manuscript. If not please clearly explain in the last paragraph of the introduction as suggested and in the methods section.

Discussion
Discussion is a confusing and quite long section. Please present the discussion section in the same order than in results. Stay the most important findings one by one. For each one, explain your finding, physiological and ecological implications, then compare your finding with previous literature.

Conclusion
Only refer to highlight your most important findings.

**Response to both referees:** Thank you very much for your valuable feedback on our paper. We followed the recommendations of the referees and shortened the manuscript substantially. We decided to focus on the behavior of the bats when feeding on the two natural flowers and removed the part about artificial flowers as we understand that presenting both parts caused some confusion and resulted in an overwhelming paper. Further, we also removed the short section about pollen that was merely intended to demonstrate the high usage of *Merinthopodium* by *Hylonycteris* throughout the year and the resulting high familiarity with these flowers, instead we refer to former papers. We also removed the apparently misleading term “feeding performance” throughout the paper and just refer to feeding efficiency, which is clearly defined in the Methods section. We adapted the rest of the paper to these changes and hopefully also cleaned up the language. We sincerely hope that this makes the paper more straightforward and easier to understand.

**7. PLOS authors have the option to publish the peer review history of their article ([what does this mean?](https://journals.plos.org/plosone/s/editorial-and-peer-review-process#loc-peer-review-history)). If published, this will include your full peer review and any attached files.**

If you choose “no”, your identity will remain anonymous but your review may still be made public.

**Do you want your identity to be public for this peer review?** For information about this choice, including consent withdrawal, please see our [Privacy Policy](https://www.plos.org/privacy-policy).

Reviewer #1: **Yes:** Melville Brockett Fenton

Reviewer #2: No

[NOTE: If reviewer comments were submitted as an attachment file, they will be attached to this email and accessible via the submission site. Please log into your account, locate the manuscript record, and check for the action link "View Attachments". If this link does not appear, there are no attachment files.]

While revising your submission, please upload your **figure files to the Preflight Analysis and Conversion Engine (PACE) digital diagnostic tool,** [**https://pacev2.apexcovantage.com/**](https://pacev2.apexcovantage.com/). PACE helps ensure that figures meet PLOS requirements. To use PACE, you must first register as a user. Registration is free. Then, login and navigate to the UPLOAD tab, where you will find detailed instructions on how to use the tool. If you encounter any issues or have any questions when using PACE, please email PLOS at [figures@plos.org](mailto:figures@plos.org). Please note that Supporting Information files do not need this step.
