# Supplementary material for: Feeding efficiency of two coexisting nectarivorous bat species (Phyllostomidae: Glossophaginae) at flowers of two key-resource plants
Source: PLoS One. 2024 Jun 26;19(6):e0303227. doi: 10.1371/journal.pone.0303227 (PMC11207168; doi:10.1371/journal.pone.0303227)
Supplement: Attachment — Submitted filename: Response to Reviewers.docx [file pone.0303227.s010.docx]

Dear Dr. Becker,

Thank you for the opportunity to revise our manuscript titled " Feeding efficiency of two coexisting nectarivorous bat species (Phyllostomidae: Glossophaginae) at flowers of two key-resource plants." We appreciate the time and effort that the reviewers have dedicated to evaluating our work, and we are grateful for their constructive feedback.

We are pleased to hear that the reviewers found our study to be generally well-executed and insightful. However, we acknowledge the concerns raised regarding the clarity of our hypothesis. Upon careful review, we have made adjustments to ensure that the hypotheses are clearly articulated and made numbered and specific predictions.

We are confident that this revision will address the reviewers' concerns and enhance the clarity of our manuscript. We appreciate the opportunity to improve our work and look forward to the possibility of its publication in PLOS ONE.

Thank you once again for your consideration.

Sincerely,

Dr. Jan Bechler
